# Perennial grass root system specializes for multiple resource acquisitions with differential elongation and branching patterns

**DOI:** 10.3389/fpls.2023.1146681

**Published:** 2023-03-17

**Authors:** Nichole Reed, Kyungdahm Yun, Eduardo A. Dias de Oliveira, Alina Zare, Roser Matamala, Soo-Hyung Kim, Miquel Gonzalez-Meler

**Affiliations:** ^1^Department of Biological Sciences, University of Illinois at Chicago, Chicago, IL, United States; ^2^School of Environmental and Forest Sciences, University of Washington, Seattle, WA, United States; ^3^Department of Electrical & Computer Engineering, University of Florida, Gainesville, FL, United States; ^4^Environmental Science Division, Argonne National Laboratory, Lemont, IL, United States

**Keywords:** functional traits, lateral root, grass, plant resources, root growth, root modeling, split-root, water acquisition

## Abstract

Roots optimize the acquisition of limited soil resources, but relationships between root forms and functions have often been assumed rather than demonstrated. Furthermore, how root systems co-specialize for multiple resource acquisitions is unclear. Theory suggests that trade-offs exist for the acquisition of different resource types, such as water and certain nutrients. Measurements used to describe the acquisition of different resources should then account for differential root responses within a single system. To demonstrate this, we grew *Panicum virgatum* in split-root systems that vertically partitioned high water availability from nutrient availability so that root systems must absorb the resources separately to fully meet plant demands. We evaluated root elongation, surface area, and branching, and we characterized traits using an order-based classification scheme. Plants allocated approximately 3/4th of primary root length towards water acquisition, whereas lateral branches were progressively allocated towards nutrients. However, root elongation rates, specific root length, and mass fraction were similar. Our results support the existence of differential root functioning within perennial grasses. Similar responses have been recorded in many plant functional types suggesting a fundamental relationship. Root responses to resource availability can be incorporated into root growth models *via* maximum root length and branching interval parameters.

## 1 Introduction

Roots, like leaves, are semiautonomous organs that respond to local environmental conditions (Doussan et al., 2003; López-Bucio et al., 2003; de Kroon et al., 2005; Lucas et al., 2018). Root systems develop functional traits that facilitate the acquisition of limiting resources and thereby increase plant fitness (Callaway et al., 2003; Liese et al., 2017; Fischer and Connor, 2018) while simultaneously affecting ecosystem functioning (Iversen, 2010; Bardgett et al., 2014; Pierret et al., 2016; Laliberté, 2017; Blume-Werry et al., 2019; Drewniak, 2019; Freschet et al., 2021). Many plant resources occur separately in the soil matrix (Zhan and Lynch, 2015; Carvalho and Foulkes, 2018), and it has been proposed that fundamental trade-offs exist within root systems between topsoil exploration and access to deeper soil layers (Lynch, 2013; Lynch, 2018; Lynch, 2019). But despite recent advances, our knowledge of root trait-functional relationships, in terms of resource capture, remains highly uncertain (Pregitzer, 2002; Carvalho and Foulkes, 2018; Freschet et al., 2021). The specialization of root systems for the acquisition of one resource can cause trade-offs for the acquisition of another (Ho et al., 2005; Lynch, 2019; Palta and Turner, 2019; van der Bom et al., 2020), and mechanisms by which root systems co-specialize for multiple resource acquisitions are not well understood (Manschadi et al., 2008; Rich and Watt, 2013). As a result, our ability to simulate plant-soil interactions and root effects on ecosystem functioning is limited in earth system models (Jackson et al., 2000; Matamala and Stover, 2013; Smithwick et al., 2014; McCormack et al., 2015; Warren et al., 2015).

The majority of research on root trait-functional relationships has capitalized on a few commonly measured “soft” traits that are widely applicable to—but have limited inferential power on—root functioning (de la Riva et al., 2021; Freschet et al., 2021). For example, rooting depth is important for water acquisition because plant access to water typically increases as roots explore deeper soil regions (Comas et al., 2013; Kitomi et al., 2015; Schneider et al., 2020), but plant nitrate acquisition also benefits (Thorup-Kristensen, 2001; Lynch, 2019; Thorup-Kristensen et al., 2020; Griffiths et al., 2022). Specific root length, another commonly-utilized trait, describes the relation between root biomass and length and is often used as a proxy for soil exploration (Freschet et al., 2021), but its relationship with branching patterns is unresolved (Glimskär, 2000; Eissenstat et al., 2015; Freschet et al., 2021). Furthermore, morphological traits are unlikely to fully represent root functioning if a single value is used to represent the entire root system (McKay Fletcher et al., 2020). If root systems are responding to multiple resources simultaneously, single measurements will encompass multiple responses.

It has been proposed that roots perform functions based on their age and relative positions within the root system (Pregitzer, 2002; Guo et al., 2008; Rewald et al., 2011; Erktan et al., 2018) and that measurements incorporating functional root classifications are more likely to increase our predictive understanding of dynamic fine root (≤ 2-mm diameter) processes. McCormack et al. (2015) suggested an order-based functional fine root classification partitioning “absorptive” from “transport” fine roots, which in woody plants can be distinguished by anatomical differences such as the presence of cork periderm or increased lignification. However, disagreements exist on the presence of differential root functioning in perennial grasses, for which anatomical evidence is more limited. Nevertheless, the assumption that all roots in cereals function identically has been repeatedly challenged (Waisel et al., 2002; Doussan et al., 2003), and an order-based classification may elucidate differential functional roles of fine roots in perennial grasses. The importance of primary roots in vertical soil exploration and of lateral roots in patch foraging and soil proliferation has been well-established for cereals (Rich and Watt, 2013) and is a key factor in crop breeding initiatives for drought avoidance (Gewin, 2010; Comas et al., 2013; Kitomi et al., 2015; Lynch, 2018) and increased nutrient capture (Lynch, 2021; Maqbool et al., 2022).

Split-root studies are commonly used to investigate localized effects of belowground environments on root systems. Many studies have identified differential branching patterns and elongation in response to heterogeneous nutrient supply (Drew et al., 1973; Gersani and Sachs, 1992; Dundabin et al., 2002; Linkohr et al., 2002; Ruffel et al., 2011; Poitout et al., 2018). Ruffel et al. (2011) described a “dormant foraging strategy” characterized by suppressed lateral root development for portions of Arabidopsis seedling root systems growing with low nitrogen (N) availability in contrast to “active foraging” in portions growing with high N availability. This response highlights lateral root elongation as a mechanism for increasing N acquisition locally (increased elongation) but also distally (reduced elongation) *via* promoting compensatory lateral root growth where N availability is high. However, the “dormant” characterization should not imply that portions of root systems growing with low nutrient availability are not actively absorbing resources. For example, water can be accessed by roots from deeper soil regions where nutrient supply is low and transported to drier soil where nutrient supply is high, thereby mobilizing nutrients and facilitating root penetration of soil while also hydrating plant tissues (Pierret et al., 2016). Reduced lateral root development can also be advantageous for water foraging because it facilitates increased rooting depth (Ho et al., 2005; Nibau et al., 2008; Lynch, 2013; Zhan et al., 2015; Yu et al., 2019) and reduced root turnover (McCormack et al., 2015; Kou et al., 2018). In split-root experiments, if suppressed lateral root development is observed for portions of root systems that are actively acquiring water but not nutrients, it suggests that reduced lateral root elongation does not compromise water acquisition and is a mechanism that plants can utilize to co-specialize for multiple resource acquisitions.

In this study, we highlight the value of measuring root traits using an order-based classification for assessing plant resource acquisition in perennial grasses. We grew *Panicum virgatum* (switchgrass) in a split-root experimental design that exposed portions of the root system to different levels of resource availability. One half of the root system was exposed to high water availability but no nutrients. The other half of the root system was exposed to low water and high nutrient availability. As plants grew, the two halves of the root systems became specialized to their respective environments, and plants were forced to acquire resources from both environments to fully meet growth demands. We utilized relatively simple measurements (root length, surface area, number of tips) but partitioned values based on the centrifugal segment root classification scheme (Berntson, 1997). We demonstrate that this approach captures variation between root system responses to resource availability and can be used to characterize root functioning in terms of resource acquisition. We reproduce our observations in a 3D root growth model to demonstrate a possible framework by which root trait-functional relationships may be incorporated into earth system models. Finally, we discuss the implications of this research for conceptual models on differential root functioning in grasses.

## 2 Material and methods

### 2.1 Root box construction and planting

We modified a root box design from the USDA National Resources Conservation Service (Trent, 2009) using plexiglass and wood wrapped in Polyguard PVC lining. Boxes were 20 × 27 × 107 cm and separated vertically by a plexiglass barrier to create two evenly sized compartments. Plant substrate was a 3:1 ratio of vermiculite to perlite mixture with a layer of stones at the base to facilitate drainage. The water holding capacity (WHC) of substrate was determined *via* gravimetric water retention curves. Boxes were tilted at a 15-degree angle to facilitate root growth against the sides of plexiglass, which were covered with tarps except when utilized for root tracing.

We collected four switchgrass plants in October 2019 from bioenergy field plantings located at Fermi National Laboratory in Batavia, IL. Plants were transported to the University of Illinois at Chicago, and aboveground tissues were removed. Plants were kept with root systems and surrounding soil intact at 5°C with no light until further processing. We washed and divided rhizomes to obtain four clones per individual with similar numbers of buds (10–11), and we trimmed roots to 5 cm. Divided rhizomes were kept in a nutrient solution composed of tap water and Hoagland’s No. 2 Basal Salt Mixture solution (Caisson Laboratories Inc.; elemental composition available in **Figure S1**) under growing lights (12 hours of light per day) for 3 days before planting to obtain observations of new growth and ensure that plants were alive. One clone was planted per box above the top of the barrier so that roots would be evenly divided between the split-root compartments. We periodically reassessed plant positions above the split-root barrier throughout the experiment, and no major shifts in rhizome positions were observed. Plants were grown indoors in winter 2019 and spring 2020 with ambient temperatures (20.2–23.7°C), CO_2_, and relative humidity (24.5–55.5%). Plants received 12 hours of light per day with an average 395-μmol m^−2^ s^−1^ PAR at canopy from Viparspectra Full Spectrum LED grow lights. Root box positions were rotated once per week.

### 2.2 Experimental treatments

Each clone received 9 L of deionized water and 59.9 mg of N once per week in the form of 350 ml of Hoagland solution (**Figure S1**), with an adjusted pH of 6–6.5. Two treatments were applied: a resource-mixed (*n* = 4) and a resource-partitioned treatment (*n* = 9). Replicates per treatment are uneven because our focus changed from 2019 to 2020 from comparing treatments to verifying the patterns we observed for the resource-partitioned treatment with additional data. From the additional eight clones grown in winter 2020 three failed to grow, resulting in nine replicates for the resource-partitioned treatment. The resource-mixed treatment consisted of both compartments receiving 1.5 L of water 3 times per week and 175 ml of Hoagland once per week. The resource-partitioned treatment consisted of one compartment receiving 3 L of water, or approximately 100% of WHC, 3 times per week and the other compartment receiving 350 ml of the Hoagland solution, or approximately 5% of WHC, once per week. This amount of Hoagland solution was used because it corresponded to the recommended 1x strength ratio of water:nutrient powder to deliver approximately 60 mg of N per dose. The goal of the resource-partitioned treatment was not to keep one half of the root system wet and the other half completely dry; water is transferred by roots from areas of high water availability to fuel growth in areas where water is absent (Boyer et al., 2010). The goal of the resource-partitioned treatment was to achieve specialization of water acquisition from one half of the root system while all nutrients must be obtained from the other half, so that root trait-functional relationships for water versus nutrient acquisition can be compared. To minimize chances of resources moving into the inappropriate compartment upon application, water and nutrient applications were not given within 8 cm of the plexiglass barrier, but they were otherwise well-distributed across substrate surfaces (1 mg of N per 5.4 cm² for resource-partitioned, 0.5 mg of N for resource-mixed). Above-ground tissues were monitored for signs of nutrient oversupply and nutrient or water deficiency throughout the experiment. Following plant harvest, substrate from water compartments was tested with an elemental analyzer (ECS 4010 CHNSO Analyzer, Costech Analytical Technologies, Inc., US) to ensure no nutrients were present.

### 2.3 Root trait measurements

We used the developmental or centrifugal segment root classification scheme (Berntson, 1997) to classify roots into orders that we term “root branching number” to avoid confusion with the centripetal classification scheme commonly utilized to distinguish root orders. For our trait analyses, primary roots originating from rhizomes were labeled as root branching number 0, and secondary roots were labeled as root branching number 1, and we similarly labeled tertiary (branching number 2), quaternary (branching number 3), and quinary roots (branching number 4).

Roots that grew against the plexiglass front were traced 3 times weekly with ultrafine colored pens and transparent sheets of plastic, which were then sprayed with an adhesive to fix the ink. Traces were scanned and analyzed in WinRHIZO Pro. The combined length of all roots together in a traced image was used to obtain elongation rates because, for small lateral roots, the presence of one individual root (as opposed to many individuals) often could not be confirmed. Absolute root elongation rates (cm day^−1^) were calculated as the change in root lengths since the last tracing divided by the number of days since the last tracing. To account for differential rates of plant growth, relative elongation rate (mm cm^−1^ day^−1^) was calculated as:


(1) Relative elongation rate=Li÷(absolute elongation rate)×10

where *L_i_* is the increase of root length (cm) in a trace recorded on the day *i*. We conducted a 20% trim (removal of the 20% highest and lowest values) on datasets of relative elongation rates before statistical modeling to avoid the incorporation of artificially high values created by equation (1).

Plants were harvested when they developed seven fully collared leaves, which is stage 7 of the Sanderson Development Index (Sanderson, 1992). Plants contained on average approximately 30 g of dry biomass at harvest. For each compartment, roots and rhizomes were washed and analyzed separately. The entirety of the root system for each clone was scanned (minus rhizomes). Primary roots with laterals still attached were scanned and root length, surface area, number of tips, and lateral branching angle measurements were collected for each root branching number (BA) using WinRHIZO. We calculated branching intensity (BI) as the total number of root tips divided by the total length of roots of the *same* branching number (Liese et al., 2017), branching density (BD) as the total number of root tips divided by the total length of roots of the *preceding* branching number (Pagès, 2019; Placido et al., 2020), and branching ratio (BR) as the total number of root tips divided by the total *number of roots* of the *preceding* branching number (Chen et al., 2013; Kong et al., 2014). After scanning, roots were dried at 65°C, weighed, and subsamples of each root branching number were prepared for chemical analysis. For each split-root compartment, we calculated the specific root length (SRL, root length divided by root biomass) and the mass fraction (root biomass in a compartment divided by total root biomass of the clone).

Roots and above-ground tissues were dried for 48 hours at 65°C before being ground and analyzed for carbon (C) and N concentrations as well as stable isotopic signatures (expressed in delta notation where N isotopic standard was atmospheric air) using an Isotope Ratio Mass Spectrometer Delta Plus XL (Thermo Finnigan, Germany). Because Hoagland solution has a high δ^15^N, isotopic analysis allows for the rudimentary tracing of N through plant tissues. There was not enough biomass in root branching number 4 for a separate chemical analysis. Due to funding constraints, no elements beyond C and N were analyzed.

### 2.4 Statistical analysis

All statistical analyses were done using R software (R Core Team, 2019). For each treatment, we used ANOVAs to test for differences in the proportions of root length, surface area, and tips allocated to each compartment. Proportions were calculated separately for each root branching number (with the exception of mass fractions) using binomial distributions. Root branching number was considered a categorical variable. For the resource-mixed treatment, we randomly selected a split-root compartment from each box to calculate proportions. For data involving multiple measurements on a single plant (BI, BD, BR, BA and SRL), we first tested for differences between plants within treatments. No differences between plants were found (**Table S1**), and so differences between treatments and compartments were also calculated using one-way ANOVAs. Generalized linear mixed-effect models were used to compare relative root elongation rates. Statistical modeling of negative binomial distributions was performed using the MASS package in R (Venables and Ripley, 2002). Generalized linear mixed-effect models were done using the glmmAMBD package (Fournier et al., 2012).

### 2.5 3D structure growth modeling

The root structure in the resource-partitioned treatment was simulated by CropRootBox.jl model, which is an adaptation of the root growth algorithm from CRootBox implemented on Cropbox modeling framework and written in Julia programming language (Schnepf et al., 2018; Yun and Kim, 2022). A set of growth parameters for the resource-partitioned treatment was obtained from data collected from the split-root compartments (**Table S2**). Axial and basal lengths of individual roots were measured using a subset of data from root scans in WinRHIZO. Lateral Branching density was calculated similarly for individual roots to proxy mean lateral branching interval (Pagès, 2019). Relative root elongation rates were obtained from root trace analysis. Branching only up to branching number 2 was assumed for the sake of simplicity. The simulation starts with an initial number of primary roots (maxB) which elongate by the elongation rate (r) and insert new lateral branches by a certain interval (ln) of length. A root segment will not start branching until it is past the axial zone (la). At the end of the segment, the basal zone (lb) is present. Each root segment keeps growing until it reaches the maximum length (lmax). An axial insertion angle of the branch is sampled from a normal distribution as indicated by the parameter (θ) whereas an angular angle is uniformly distributed. The subsequent lateral roots follow a similar pattern of growth as the primary root but are controlled by a separate set of parameters assigned for each root branching number. A virtual root structure was generated 100 times with a randomly sampled set of parameters. An average total root length per branching number (cm) was then calculated for comparison with actual measurements.

## 3 Results

**Figure 1:**
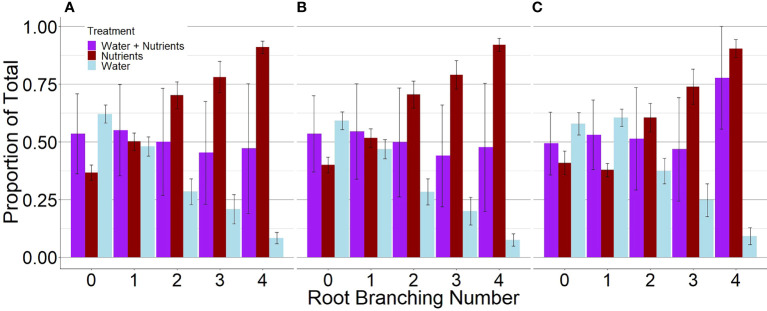
Proportions of root length **(A)**, surface area **(B)**, and tips **(C)** of *P. virgatum* allocated towards each compartment type by root branching number (mean ± SEM). “Water + Nutrients” represent split-root compartments in the resource-mixed treatment. To analyze proportions, one split-root compartment was randomly selected from each box. The “Nutrients” and “Water” represent split-root compartments in the resource-partitioned treatment where water was limited but nutrients were available and where no nutrients were present but water availability was high, respectively. Allocation to higher root branching numbers represent greater lateral root development.

**Figure 2:**
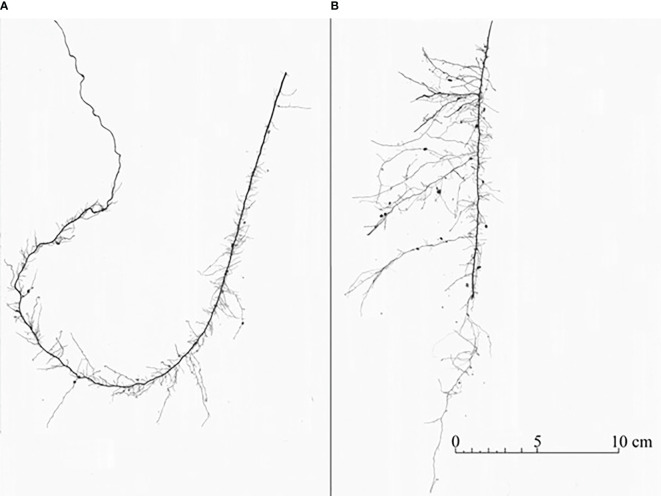
Roots from resource-partitioned treatment growing with high water **(A)** and nutrient **(B)** availability from the same individual of *P. virgatum*. Roots acquiring only water **(A)** typically exhibited greater elongation of primary roots and reduced development of lateral branches relative to roots growing with nutrients **(B)**.

*P. virgatum* elongated lateral roots in response to nutrient availability but elongated primary roots in response to high water availability (**Figures 1**, **2**). For plants grown with resources evenly mixed, the length, surface area, and the number of tips of branching numbers 0–3 were equally distributed between both compartments. In root branching number 4, root length and surface area were evenly distributed but root tips were not; however, differences were not statistically significant (P = .92). For the resource-partitioned treatment, the proportion of root length, surface area, and number of tips allocated towards water acquisition ranged from 59–63% in branching number 0 to 8–10% in branching number 4 (**Table S3**). Conversely, the proportion of root length allocated towards nutrients increased with increasing branching number. The length and surface area of root branching number 1 were distributed evenly between compartments, but no other root branching number exhibited even distribution in the resources-partitioned treatment. This pattern was particularly evident in root branching number 3, where the estimated proportions of root length, surface area, and tips were 90% lower in water than in nutrient compartments (P = .02 for length and surface area, P = .05 for tips), and for branching number 4 where estimates were 99% lower (P = .005 for all).

Branching intensity (BI), branching density (BD), branching ratio (BR), and lateral branching angle (BA) all varied between root branching numbers (P <.001, **Table 1**). In the resource-partitioned treatment, BI was greater in roots from water compartments compared to nutrient (P = .004), with model estimates 68% higher for branching number 1 and 95% higher for 2 (P = .001 and .003, respectively; **Table S4**). However, there were no significant differences for BD. Branching ratio for roots growing with high water availability was nearly 60% lower than for roots growing with nutrients for branching number 2 (P <.001), and BA for branching number 1 was smaller in roots growing with nutrients (P = .017). In the resource-mixed treatment, BI, BD, BR and BA did not differ between compartments for any root branching number. Specific root length and mass fraction did not vary significantly between treatments or compartments (**Tables 2**, **S5**). Relative elongation rates also did not differ in either the resource-mixed (P = .91) or resource-partitioned treatments (P = .33).

**Table 1:** Branching intensity (tips cm^−1^), branching density (tips cm^−1^), branching ratio (tips roots^−1^), and lateral branching angles (degrees) by branching number of *P. virgatum* for roots growing in three compartment types (mean ± SEM).

Compartment	Branching Number	Branching Intensity	Branching Density	Branching Ratio	Branching Angle
**Nutrients**	0	0.2 ± 0.0	n/a	n/a	n/a
	1	1.0 ± 0.1	5.0 ± 0.5	45.1 ± 6.7	48.2 ± 0.9
	2	1.9 ± 0.2	0.8 ± 0.2	1.4 ± 0.2	55.5 ± 1.3
	3	2.6 ± 0.3	0.4 ± 0.1	0.4 ± 0.1	60.8 ± 1.4
	4	1.8 ± 0.3	0.2 ± 0.0	0.2 ± 0.1	61.8 ± 1.2
**Water**	0	0.1 ± 0.0	n/a	n/a	n/a
	1	1.7 ± 0.1	6.1 ± 0.3	47.8 ± 5.1	53.0 ± 1.6
	2	3.8 ± 0.5	1.3 ± 0.1	0.6 ± 0.1	59.3 ± 1.8
	3	3.9 ± 0.8	0.6 ± 0.2	0.2 ± 0.1	60.9 ± 1.2
	4	2.5 ± 0.8	0.5 ± 0.1	0.2 ± 0.1	62.3 ± 1.3
**Resource-** **mixed**	0	0.1 ± 0.0	n/a	n/a	n/a
	1	1.5 ± 0.3	5.8 ± 0.3	40.1 ± 5.4	51.5 ± 2.1
	2	3.7 ± 0.9	0.9 ± 0.2	0.7 ± 0.2	55.4 ± 2.7
	3	4.9 ± 1.9	0.5 ± 0.1	0.2 ± 0.1	65.3 ± 3.3
	4	2.7 ± 0.7	0.5 ± 0.2	0.1 ± 0.0	61.4 ± 4.3

The “Nutrients” and “Water” represent split-root compartments in the resource-partitioned treatment where water was limited but nutrients were available and where no nutrients were present but water availability was high, respectively.

**Table 2:** Specific root length (SRL), mass fraction of root biomass, and relative root elongation rates of *P. virgatum* present in three compartment types (mean ± SEM).

Compartment	SRL (cm g^−1^)	Mass fraction (g g^−1^)	Relative elongation rate (mm cm^−1^ day^−1^)
**Nutrient**	3702 ± 777	.50 ± .04	1.14 ± 0.1
**Water**	3252 ± 607	.50 ± .04	1.01 ± 0.1
**Resource-mixed**	4845 ± 805	.56 ± .20	1.01 ± 0.1

Mass fraction for the resource-mixed treatment was determined by randomly selecting one split-root compartment from each root box.

Root % N did not differ between root branching numbers (P = .83 for resource-mixed, P = .51 for resource-partitioned), but root C:N ratios in the resource-partitioned treatment did (P = .018) with root branching numbers 3 and 4 being lower than others (**Table 3**). Root % N was lower for roots in the resource-partitioned treatment than in resource-mixed (P = .02), but within both treatments there was no effect of compartment on root % N or C:N ratios (**Table S6**). In the resource-partitioned treatment, mean root δ^15^N was 52% lower in roots growing with high water availability compared to roots growing with nutrients (P <.001, **Table S6**). For the resource-mixed treatment, there was no difference. Above-ground % N and C:N ratios did not vary between treatments, but δ^15^N varied for above-ground tissues (P = 0.03).

**Table 3:** Nitrogen (N) and Carbon (C) content (%), C:N ratios, and δ^15^N (‰) of *P. virgatum* root tissues, separated by branching number as well as total above-ground plant tissues (mean ± SEM).

Type	Root Branching Number	% N	% C	C:N ratio	δ^15^N
Nutrients	0	0.7 ± 0.1	46.7 ± 0.6	117 ± 45	3.7 ± 0.9
	1	0.7 ± 0.2	42.2 ± 2.4	200 ± 72	5.0 ± 1.9
	2	0.8 ± 0.2	35.2 ± 4.2	66 ± 24	8.1 ± 4.9
	3 & 4	0.8 ± 0.1	37.2 ± 3.2	43 ± 9	7.2 ± 3.0
Water	0	0.6 ± 0.1	46.0 ± 1.4	91 ± 20	2.8 ± 2.5
	1	0.7 ± 0.2	41.8 ± 1.8	91 ± 47	3.5 ± 1.8
	2	0.6 ± 0.2	40.6 ± 2.4	135 ± 42	2.6 ± 1.4
	3 & 4	0.7 ± 0.2	26.7 ± 5.5	51 ± 21	3.3 ± 1.6
Resource-mixed	0	1.0 ± 0.3	45.7 ± 1.1	72 ± 20	3.0 ± 1.2
	1	1.3 ± 0.2	39.8 ± 3.0	41 ± 9	3.4 ± 1.1
	2	1.3 ± 0.2	41.3 ± 1.7	38 ± 9	3.0 ± 1.3
	3 & 4	1.0 ± 0.01	39.1 ± 7.0	41 ± 8	2.9 ± 1.7
**Resource-mixed above ground**		1.5 ± 0.3	45.0 ± 1.0	59 ± 22	2.7 ± 0.4
**Resource-partitioned above ground**		1.4 ± 0.1	44.6 ± 0.8	51 ± 6	4.2 ± 1.2

**Figure 3:**
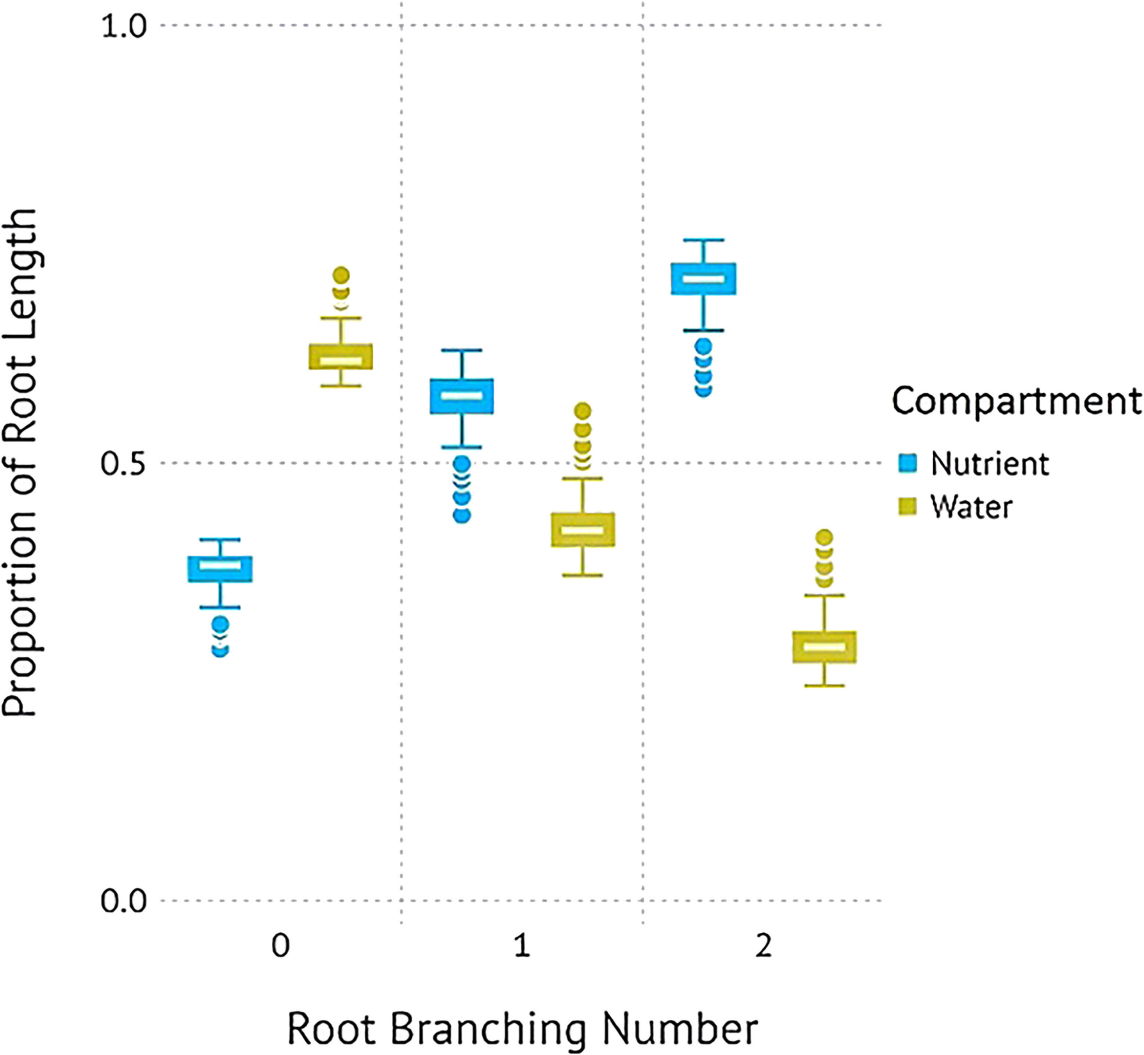
Proportions of root length for *P. virgatum* simulated with the 3D root structure CropRootBox.jl model parameterized for the resource-partitioned treatment in an attempt to replicate **Figure 1A**.

**Figure 4:**
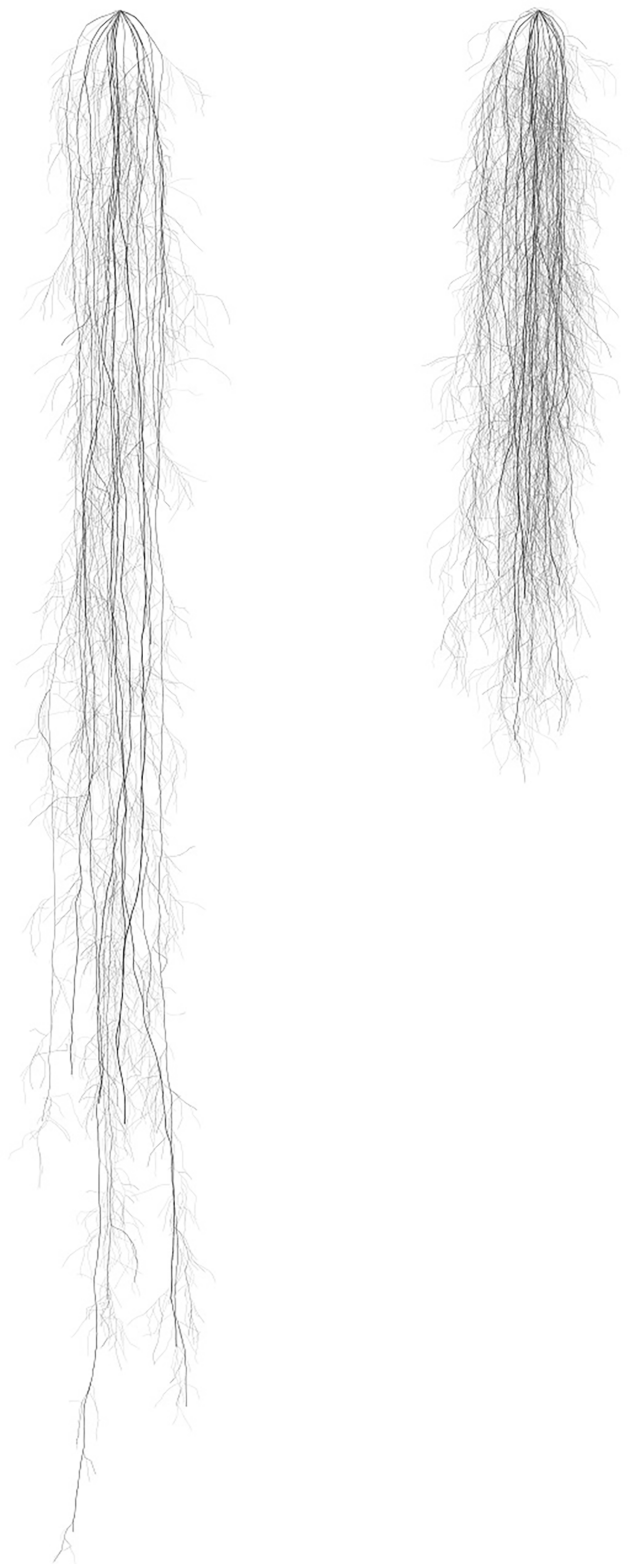
A rendering of 3D root structure modeled by CropRootBox.jl using the growth parameters obtained from roots growing with high water availability (left) and roots growing with nutrient availability (right) in the resource-partitioned treatment.

Simulations with the 3D root model showed a similar result to in **Figure 1**. When comparing the total root length per root branching number, root architecture developed under high water and no nutrient availability had a similar total length and pattern of divergence with subsequent branching of lateral roots. Similar to **Figure 1A**, root systems parameterized for the nutrient compartments had a significantly larger length of lateral roots compared to those growing in the water compartments (**Figure 3**). A visual comparison of root systems in 3D rendering also confirmed vigorous growth of lateral roots in nutrient compartments with less rooting depth and more fine-grained lateral roots (**Figure 4**).

## 4 Discussion

In this study, we exposed *P. virgatum* root systems to environments that differed in water and nutrient availability in an effort to demonstrate root trait-functional relationships for resource acquisitions. For plants in the resource-partitioned treatment, nutrients were only available to half of the root system, but plants were also required to absorb water where no nutrients were present in order to fully meet plant growth demands and to transport water to dry substrate. The progressive increase in lateral root length, surface area, and tips allocated towards dry, nutrient-rich compartments indicates that differential elongation of lateral roots is an essential mechanism for co-optimizing multiple resource acquisitions in *P. virgatum* (**Figure 1**). The short lateral branches (mostly root branching number 1) associated with high water availability, but nutrient absence, may have facilitated passive uptake of water through lateral root tips (Ahmed et al., 2016). Root tips were previously highlighted in a study of grapevine that reported the majority of water absorption in drought-resistant rootstocks occurred surrounding tips during soil rewetting (Cuneo et al., 2021). Increased elongation of primary roots has also been associated with water acquisition in other studies (Zhan et al., 2015) and is a common response to water deprivation in nature (Dinney, 2019; Calleja-Cabrera et al., 2020; Maqbool et al., 2022). In contrast, allocating 70% or more of root surface area in branching numbers 2–4 towards nutrients indicates that plants optimized nutrient uptake by maximizing lateral root surface area. This response has been observed before and facilitates greater nutrient uptake, especially when nutrients are relatively immobile (Nye and Tinker, 1977; Zhan and Lynch, 2015).

Traits that encompassed root topology were able to capture variation between below-ground environments. In the resource-partitioned treatment, water compartments had a moderately positive effect on branching intensity (BI, **Table S4**) because decreased root lengths increased BI for branching numbers 2 and 3. Also, the decreased branching ratio (BR) for root branching number 2 in water compartments reflected the reduced tertiary root development that occurred in the absence of nutrients. However, branching density (BD), which is perhaps the most widely used measurement of the three, was not as effective in describing differential root responses. Water availability triggered emergence of root branching number 1 from primary roots, which has also been observed in hydropatterning studies (Bao et al., 2014; Giehl and von Wirén, 2018); however, we did not observe widespread emergence of root branching numbers 3 or 4 in the absence of nutrients (**Table S3**). Thus, our results question the relevance of hydropatterning for higher branching numbers. The role of hydropatterning in fibrous root systems has not been thoroughly investigated and requires further study before mechanisms can be confirmed.

Studies of architectural development are limited by intensive labor requirements and poor understanding of below-ground root distribution across different environments. But the development of root system architecture can be simulated with computational models with a varying degree of detail in the representation of root structure. Our CropBox model is a three-dimensional (3D) root architecture model that explicitly describes individual roots with 3D geometric shapes and allows for the comparison of morphological traits between phenotypes (Dunbabin et al., 2013; Postma et al., 2017; Schnepf et al., 2018). The incorporation of root responses to high water versus nutrient availability was possible *via* root branching and length parameters. Specifically, the model utilized branching intervals (ln) and maximum root lengths (lmax) to recreate differential elongation of root branching numbers 0–2 (**Table S2**). Furthermore, although we observed minimal differences in elongation rates (**Table 4**), our observations were limited to a fraction of the total roots (only roots growing against the plexiglass sides of the boxes were traced). Further applications of root growth models may elucidate the importance of elongation rates, particularly of different root branching numbers, for optimal resource acquisition. Finally, 3D root growth models can provide information on dynamic root processes that can be utilized in earth system models to decrease uncertainty associated with below-ground responses to changing climate and environments (Bassu et al., 2014; Drewniak, 2019; Berkelhammer et al., 2022).

**Table 4:** Absorptive root elongation rate (mm cm^-1^ day^-1^) and transport root elongation rate (mm cm^-1^ day^-1^), overall root elongation rate (mm cm^-1^ day^-1^) and growth rate (mg g^-1^ day^-1^), absolute total root elongation (cm day^-1^), and root biomass (mg) of *P. virgatum* for nutrient, water, and water + nutrient compartments. Root elongation and growth rates are 20 % trimmed mean ± SEMs.

Parameter	Nutrients	Water	Water + Nutrients
**Root elongation rate**	1.14±0.1	1.01±0.1	1.01±0.1
**Root growth rate**	113±10	102±10	101±8
**Absorptive root elongation rat**	2.04±0.92	1.40±0.31	1.73±0.40
**Transport root elongation rate**	1.12±0.10	0.98±0.10	0.97±0.08
**Root biomass**	0.97±0.3	0.63±0.1	0.27±0.1
**Avg. total root elongation**	43.7±14.8	37.5±8.9	17.1±4.2

*P. virgatum* distinguished between high water versus nutrient availability by elongating different root branching numbers (**Figure 1**). McCormack et al. (2015) proposed that the most distal fine roots are absorptive and are responsible for the majority of resource uptake, while fine roots that branch extensively and undergo secondary development are transport roots through which water and nutrients are distributed but in which limited uptake occurs. In this context, plants can prioritize either transport or absorptive root elongation depending on the soil environment (*E.g.*, Dunbabin et al., 2001; Williamson et al., 2001). Despite the lack of anatomical evidence of differential root functioning in non-woody plants, such as secondary development, many dynamics similar to those described for absorptive and transport fine roots exist within non-woody root systems. For example, primary fine roots in perennials persist longer than laterals (Liu et al., 2016), similar to transport fine roots in woody plants (Xia et al., 2010; McCormack et al., 2015). Fine root production represents a significant photosynthate investment (Lambers et al., 2002; Lynch, 2013; Palta and Turner, 2019). But, once longer-lasting transport roots are established, absorptive fine roots can be invested at any time that resources become available (Zhang et al., 1999; Dunbabin et al., 2001; Malamy, 2005). If adequate water absorption can be achieved through secondary root tip emergence alone, continued development of higher root branching numbers for water acquisition is an unnecessary use of internal plant resources (Lynch, 2019). While more research is needed on the role of lateral root development for water absorption in *P. virgatum*, our study does suggest that differential root functioning is present in perennial grasses.

Our study includes numerous caveats, a few of which are listed here. First, recreating root architecture and morphology as it occurs in soil is beyond the scope of this study. A vermiculite:perlite mixture does not have the chemical properties of soil and has different water holding and release properties, and this can alter nutrient dynamics. Our study also does not address responses of plants to interspecific competition, which has profound effects on root growth (McNickle and Dybzinski, 2013), or represent a global plant response to water and nutrient availability. In some environments, elongation of lateral roots is a more viable strategy for water acquisition (Moreira et al., 2000; Cheng et al., 2006). Second, for the resource-partitioned treatment, the lack of moisture may have contributed to reduced root diameters and ultimately caused the underestimation of root surface area in nutrient compartments. Dry conditions may have also led to an underestimation of absolute root elongation rates for roots growing with nutrients (Boyer et al., 2010).

Root length, surface area, and the number of tips all varied significantly between resource-partitioned split-root compartments but only when separated by branching number (**Tables S3**, **S5**). Below-ground measurements of root systems that do not account for differential responses of root branching numbers will not fully capture plant responses to resource availability, as shown in our measurements of SRL and mass fraction (**Table 2**). By restricting plants’ access to water, we have shown that decreased lateral root development in nutrient-poor regions is not merely a side effect of increased development in nutrient-rich regions, but it is also a viable strategy for maximizing water absorption. The model simulation explained differences in root system development using a small number of traits, notably greater maximum root lengths and reduced branching intervals, that vary between root branching numbers. Parameterization of root responses to resource availability in root growth models helps inform experiments, but it can also provide a link between physiological models and simulations of below-ground processes in earth system models *via* information on carbon assimilation and root turnover (i.e., Lynch et al., 2013). Advances in root phenotyping that enable higher throughput, such as automated analysis of root imaging, can enhance root architecture understanding and alleviate some labor-intensive aspects of studying roots (Xu et al., 2020; Yu et al., 2020a; Yu et al., 2020b; Xu et al., 2022). Studies that explore differential responses of root branching numbers will further elucidate the mechanisms plants utilize to co-optimize for multiple resource acquisitions and will increase our understanding of plant and ecosystem functioning.

## Data availability statement

The raw data supporting the conclusions of this article will be made available by the authors, without undue reservation.

## Author contributions

This research was designed by NR, KY, ED, AZ, S-HK, and MG-M. Statistical analyses were performed by NR and KY. The manuscript was drafted by NR and KY and edited by ED, AZ, RM, S-HK, and MG-M. All authors contributed to the article and approved the submitted version.
