# Supplementary material for: Perennial grass root system specializes for multiple resource acquisitions with differential elongation and branching patterns
Source: Front Plant Sci. 2023 Mar 17;14:1146681. doi: 10.3389/fpls.2023.1146681 (PMC10064013; doi:10.3389/fpls.2023.1146681)
Supplement: Supplementary file 1 [file DataSheet_1.pdf]

Supplementary Material

PERENNIAL GRASS ROOT SYSTEM SPECIALIZES FOR MULTIPLE RESOURCE ACQUISITIONS WITH DIFFERENTIAL ELONGATION AND BRANCHING PATTERNS

Nicholas T Glass, Kyungdahm Yun, Eduardo A. Dias de Oliveira, Alina Zare, Roser Matamala, Soo-Hyung Kim, Miquel Gonzalez-Meler*

*** Correspondence:** Corresponding Author: mmeler@uic.edu

Components	mg/L
Ammonium Phosphate, Monobasic ($NH_4H_2PO_4$)	115.0300
Boric Acid (H_3BO_3)	2.8600
Calcium Nitrate, Tetrahydrate ($Ca(NO_3)_2 . 4H_2O$)	656.4000
Cupric Sulfate, Pentahydrate ($CuSO_4 . 5H_2O$)	0.0800
Ferric Tartrate ($C_{12}Fe_2H_{12}O_1 8$)	5.3200
Magnesium Sulfate, Anhydrous ($MgSO_4$)	240.7600
Manganese Chloride, Tetrahydrate ($MnCl_2 . 4H_2O$)	1.8100
Molybdenum Trioxide (MoO_3)	0.0200
Potassium Nitrate (KNO_3)	606.6000
Zinc Nitrate, Hexahydrate ($Zn(NO3)_2 . {}_6H_2O$)	0.2200

Supplementary Figure 1. Elemental composition of Hoagland’s No. 2 Basal Salt Mixture solution (Caisson Laboratories Inc.).

Supplementary Table 1. F-values and *P*-values for effects on branching intensity (BI), branching density (BD), branching ratio (BR), lateral branching angle (BA), and specific root length (SRL) from plants. Degrees of freedom for error was 7 for resource-partitioned and 2 for resource-mixed.

**Treatment**	**Trait**	**F-value**	***P***
Resource-mixed	BI	0.6	.7
	BD	0.5	.7
	BR	2.7	.2
	BA	5.8	.06
	SRL	1.0	.5
Resource-partitioned	BI	1.0	.5
	BD	2.0	.2
	BR	0.7	.7
	BA	2.7	.08
	SRL	2.3	.1

Supplementary Table 2. Root structure growth model parameters for water versus nutrient split-root compartments in the resource-partitioned treatment.

Symbol	Water Value	Nutrient Value	Units	Description
**Root architecture**				
**minB**	5	5	-	Minimum number of primary roots
**maxB**	22 ± 14	24 ± 18	-	Maximum number of primary roots
**Root branching number 0**				
**ln**	10 ± 1	5 ± 0.05	cm	Branching interval
**lmax**	141.59 ± 42.91	79.93 ± 15.18	cm	Maximum length of the root
**θ**	60 ± 6	60 ± 6	°	Branching angle
**a**	0.45 ± 0.26	0.46 ± 0.27	mm	Diameter
**Root branching number 1**				
**ln**	0.59 ± 0.03	1.0 ± 0.10	cm	Branching interval
**lmax**	11.01 ± 3.34	13.79 ± 2.62	cm	Maximum length of the root
**θ**	53.0 ± 1.6	48.2 ± 0.9	°	Branching angle
**a**	0.14 ± 0.11	0.15 ± 0.10	mm	Diameter
**Root branching number 2**				
**ln**	0.26 ± 0.03	0.53 ± 0.06	cm	Branching interval

**lmax**	5.97 ± 1.81	7.51 ± 1.43	cm	Maximum length of the root
**θ**	59.3 ± 1.8	55.5 ± 1.3	°	Branching angle
**a**	0.09 ± 0.07	0.10 ± 0.07	mm	Diameter

Supplementary Table 3. Binomial generalized linear model estimates and 95 % confidence intervals (CI) for proportions of total root length, surface area, and tips allocated to water compartments versus nutrients in the resource-partitioned treatment. Estimates represent proportions allocated towards high water availability. Intercept represents the nutrient compartment mean. Degrees of freedom for error was 16 for all models.

Proportion	Root Branching Number	Intercept	Water Estimate	95 % CI	z-value	*P*
**Length**	0	.37	.74	.30–.96	1.6	.3
	1	.50	.48	.12–.86	-0.1	.9
	2	.70	.14	.02–.54	-1.7	.09
	3	.78	.07	.01–.37	-2.3	.02
	4	.91	.01	.00–.12	-2.8	.005
**Surface Area**	0	.40	.69	.25–.94	0.81	.4
	1	.52	.45	.11–.84	-0.2	.8
	2	.70	.14	.02–.53	-1.7	.08
	3	.79	.06	.01–.35	-2.3	.02
	4	.92	.01	.00–.11	-2.8	.005
**Tips**	0	.41	.67	.24–.93	0.7	.5
	1	.38	.72	.28–.95	1.0	.3
	2	.61	.28	.05–.71	-1.0	.3

	3	.74	.10	.01–.46	-2.0	.05
	4	0.9	0.01	.00–.13	-2.8	.005

Supplementary Table 4. Linear and Gamma generalized linear model estimates and 95 % confidence intervals (CI) for compartment effect on branching intensity (BI), branching density (BD), branching ratio (BR), and lateral branching angle (BA) for roots in the resource-partitioned treatment. Estimates represent the differences between means. Intercept represents the nutrient compartment.

Trait	Root Branching Number	Intercept	Water Estimate	95 % CI	T-value	*P*	D.F. for Error
**BI**	0	0.16	-0.03	-0.10–0.04	-0.8	.4	16
	1	1.00	0.69	0.36–1.01	4.4	.001	16
	2	1.94	1.83	0.88–2.99	3.5	.003	16
	3	2.63	1.31	-0.10–3.01	1.7	.1	15
	4	1.78	0.72	-0.56–2.62	1	.3	12
**BD**	1	6.13	-0.37	5.48–6.78	-0.9	.4	16
	2	1.33	-0.42	1.02–1.63	-2.1	.06	16
	3	0.64	-0.12	0.41–1.07	-0.6	.6	15
	4	0.51	0.02	0.32–0.89	0.1	.9	12
**BR**	1	45.1	2.7	-15.2–20.6	0.3	.8	16
	2	1.4	-0.8	-1.2– -0.5	-4.5	<.001	16
	3	0.4	-0.2	-0.5–0.03	-1.6	.1	15
	4	0.2	0.02	-0.2–0.4	0.2	.9	12
**BA**	1	48.2	4.8	1.0–8.6	2.7	.02	16
	2	55.6	3.7	-1.1–8.5	1.6	.1	16

	3	60.8	1.9	-3.1–7.0	0.7	.5	16
	4	61.9	3.2	-5.2–11.9	0.7	.5	15

Supplementary Table 5. Linear model (SRL, resource-mixed), Gamma (SRL, resource-partitioned) and Binomial (Mass fraction) generalized linear model estimates and 95 % confidence intervals (CI) of specific root length (SRL) and mass fraction for roots in the resource-mixed and resource-partitioned treatments.

**Treatment**	**Trait**	**Intercept**	**Estimate**	**95 % CI**	**F/z-value**	***P***
Resource-mixed	SRL	3702	-4450	-2617–1585	0.2	.7
	Mass fraction	-0.23	0.47	-2.36–3.45	0.2	.7
Resource-partitioned	SRL	4242	1208	-2875–5291	0.5	.5
	Mass fraction	-0.003	0.005	-1.88–1.89	0.01	.99

Supplementary Table 6. Gamma generalized linear model (resource-mixed $\delta^{15}N$) and linear model estimates and 95 % confidence intervals (CI) for effects on chemical content of below-ground tissues from compartments in the resource-mixed and resource-partitioned treatments. Degrees of freedom for error is 6 for resource-mixed models and 16 for resource-partitioned models.

Treatment	Content	Intercept	Estimate	95 % CI	T-value	*P*
Resource-mixed	**Percent N**	1.1	-0.02	-0.86–0.82	-0.06	.95
	**C:N ratio**	60.5	-10.4	-45.8–25.1	-0.7	.5
	**$\delta^{15}N$**	2.7	0.6	-0.6–1.8	0.97	.4
Resource-partitioned	**Percent N**	0.7	-0.03	-0.3–0.2	-0.3	.8
	**C:N ratio**	108.1	-9.6	-78.5–59.3	-0.3	.8
	**$\delta^{15}N$**	5.7	-3.0	-4.4– -1.5	-4.3	<.001

Supplementary Table 7. Linear model estimates and 95 % confidence intervals (CI) for treatment effects on chemical content of above-ground tissues in resource-partitioned plants vs. resource-mixed plants. Estimates represent differences between means. Intercepts represent means of resource-mixed plants.

Content	Intercept	Resource-partitioned estimate	95 % CI	T-value	*P*	D.F. for Error
**Percent N**	1.5	-0.03	-0.59–0.54	-0.1	.9	10
**C:N ratio**	58.8	-7.6	-45.5–30.3	-0.5	.7	10
**$\delta^{15}N$**	2.7	1.5	0.2–2.8	2.5	.03	11
**$\delta^{13}C$**	-15.2	0.4	-0.03–0.9	2	.07	11
